# Evaluating the role of SARS-CoV-2 target genes based on two nucleic acid assay kits

**DOI:** 10.3389/fpubh.2022.982171

**Published:** 2022-09-28

**Authors:** Xuetong Zhu, Fengyan Zhou, Qi Zhou, Jiancheng Xu

**Affiliations:** ^1^Center of Infectious Diseases and Pathogen Biology, The First Hospital of Jilin University, Changchun, China; ^2^Department of Laboratory Medicine, The First Hospital of Jilin University, Changchun, China; ^3^Department of Infectious Disease Control, Jilin City Center for Disease Control and Prevention, Jilin, China; ^4^Department of Pediatrics, The First Hospital of Jilin University, Changchun, China

**Keywords:** coronavirus disease 2019, severe acute respiratory syndrome coronavirus 2, antigen rapid diagnostic test, reverse transcription-quantitative polymerase chain reaction, nucleocapsid gene

## Abstract

**Background:**

Effective isolation and early treatment of coronavirus disease 2019 (COVID-19) relies on rapid, accurate, and straightforward diagnostic tools. In response to the rapidly increasing number of cases, reverse transcription-quantitative polymerase chain reaction (RT-qPCR) assays for multiple target genes have become widely available in the market.

**Methods:**

In total, 236 COVID-19 patients with positive results in both RT-qPCR and rapid antigen diagnosis (Ag-RDT) were enrolled in the study. The cycle threshold (Ct) was compared with different onset times and target genes. Comparison between groups was evaluated with the Kruskal-Wallis test and Dunn test. The correlation between target genes was analyzed by Spearman.

**Results:**

In samples of Ct ≤ 21, Ct was different for the nucleocapsid (N), open reading frame 1ab (ORF1ab), and envelope (E) genes (*P* < 0.05). Mild COVID-19 patients within 7 days of onset accounted for 67.80% of all enrolled patients. At the above stage, all target genes reached the trough of Ct, and N genes showed lower values than the other target genes. The Ct of the ORF1ab and N gene in asymptomatic patients differed from those of mild patients within 7 days and more than 14 days of onset. The kits used in the study showed strong consistency among target genes, with all correlation coefficients >0.870.

**Conclusion:**

RT-qPCR confirmed that the N gene performed well in Ct ≤ 21 and samples within 7 days of onset. Ag-RDT was discriminatory for patients within 7 days of onset. This study facilitated early identification and control of COVID-19 prevalence among patients.

## Introduction

Coronavirus disease 2019 (COVID-19) is defined as an infectious disease caused by the severe acute respiratory syndrome coronavirus 2 (SARS-CoV-2). Currently, SARS-CoV-2 shows a pandemic trend, which plays a vital impact on global public health and the economy, etc. The ability of SARS-CoV-2 to spread rapidly in the absence of symptoms confirms the importance of infection surveillance efforts to control the pandemic (1). Early detection and diagnosis of COVID-19, in addition to contact tracing, isolation, and vaccination, is the first protective barrier against COVID-19 (2). An effective identification program that meets rapid, reliable, and affordable criteria can help facilitate the isolation of potentially infected patients, reduce the rate of social transmission, implement targeted treatments, and protect healthcare systems from disease transmission.

To date, probe-based reverse transcription-quantitative polymerase chain reaction (RT-qPCR) remains the gold standard for testing RNA extracted from upper respiratory tract specimens for the diagnosis of COVID-19 (3). In practical applications, RT-qPCR detection performance not only places high demands on laboratory resources, equipment, detection reagents, detection time, operators, and operating systems, but also on different stages of the disease, collection methods, and kit performance (4, 5). In view of the above, potent kit performance and efficient target gene selection facilitate large-scale regional screening when targeting deeply infectious viruses. Although the World Health Organization has recommended antigen rapid diagnostic test (Ag-RDT) as an auxiliary method for the early diagnosis of COVID-19, it is only a supplementary method for screening specific populations, which is conducive to improving the ability of early detection (6). Primary health care institutions with nucleic acid testing capabilities should prefer nucleic acid testing.

The RT-qPCR diagnostic strategy for SARS-CoV-2 mainly focuses on the N, ORF1ab, and E genes in practical applications. E gene was recommended by the World Health Organization for initial screening (7). The N gene is the most frequently selected target gene apart from ORF1ab (8). The “Guidelines for the implementation of total 2019-nCoV nucleic acid detection (2nd edition)” issued by the National Health Commission of the People's Republic of China clarified the basic qualification requirements for SARS-COV 2 detection reagents (9). Considering the above, the Novel Coronavirus 2019-nCoV Nucleic Acid Detection Kits produced by Maccura Biotechnology Co (Maccura kit) and Sansure Biotechnology Co (Sansure kit) have been chosen for this study. The Maccura kit has three targets for SARS-CoV-2, namely, the ORF1ab, N, and E genes. The Sansure kit has two targets for SARS-CoV-2, namely, the ORF1ab and N genes. Maccura kit is used for the preliminary screening of SARS-CoV-2, as its lower detection limit meets the requirements of ≤ 500 copies/ml. For Sansure, it is regarded as a retest reagent when the nucleic acid is initially screened positive because its analytical sensitivity meets the requirement of 100 to 300 copies/mL.

At present, there are many different SARS-CoV-2 RT-qPCR kits on the market for the prevention and control of the COVID-19 pandemic, providing an important guarantee for the diagnosis of COVID-19. This study aimed to clarify the influence of different onset times, target genes, and kit manufacturers on the determination of SARS-CoV-2 Ct value in patients with positive antigen screening and nucleic acid testing. In addition, the research divided the infected population according to the Ct and infection time, respectively, and identified the optimally expressed target genes and the correlation between each target gene.

## Materials and methods

### Study population

This retrospective study was conducted from March 30 to April 4, 2022. During this period, a new outbreak with a substantial increase of COVID-19 occurred in Jilin Province. Ag-RDT self-test procedures were performed by personnel in one of the following situations: (a) symptoms of respiratory infection; (b) exposure to SARS-CoV-2 positive persons; (c) home isolated persons. Patients with positive Ag-RDT test were included in the next stage of nucleic acid testing, and the selected research objects are patients with positive nucleic acid and antigen tests. Laboratory and clinical data (age, sex, time from symptom onset) of all patients were extracted from medical records. There were no specific requirements for the age and sex of the study subjects. All existing test data were retrospectively analyzed.

### Diagnostic techniques

RNA amplification was performed by Maccura kit and Sansure kit. According to Maccura's manufacturer recommendations, samples with Ct ≤ 38 for three or two gene targets are defined as positive, while Ct > 38 is negative. Sansure identified nucleic acid positives according to the following criteria: samples with Ct ≤ 40 for three or two gene targets is defined as positive, while Ct > 40 is negative. The RNA extraction procedure was performed by the automatic nucleic acid extraction instrument SSNP-9600A (Jiangsu Bioperfectus Technologies Co., Ltd.) and its supporting nucleic acid extraction/purification reagent (batch number 20210935) with a strict process. The Nucleic acid amplification instrument adopts the RT-qPCR detection system SLAN-96P (Shanghai Hongshi Medical Technology Co., Ltd.).

Regarding Ag-RDTs, the antigen of the N protein on SARS-CoV-2 was detected by the iHealth COVID-19 Antigen Rapid Test (iHealth Labs Inc.). The determination and interpretation of the self-test specimen collection procedure follow the manufacturer's instruction manual. To begin the Ag-RDT, a self-collected nasal swab is inserted into a test tube along with the reaction solution, and the interaction of the liquid in the test tube with the sample helps expose viral antigens to the antibodies used in the test. Then, add the sample fluid to the sample port of the COVID-19 test card. Results were visually interpreted after 15 min of reaction. If the extracted sample contains SARS-CoV-2 antigen, a pink T line and C line will appear on the COVID-19 test card, indicating a positive result. Line C only appears if the SARS-CoV-2 antigen is absent or at low levels.

### Statistical analyses

Categorical variables were expressed as proportions and continuous variables as the median and interquartile range (IQR). Comparison of differences between multiple groups was evaluated using the Kruskal-Wallis test and then Dunn test was used for the pairwise comparison of groups with differences. The correlation between target genes were analyzed by Spearman. Data was collected using Microsoft Excel Version 2016 (Microsoft Corporation). Statistical analyses were performed using R 4.2.3 (R Core Team).

### Quality control

Sample collection and assays of RT-qPCR were performed by trained medical staff for standardized procedures and traceability of sample results. Nucleic acid samples were stored at 2–8°C and then transported to the laboratory within 4 h after collection. The PCR reaction system configuration, reaction parameters, and program settings were all carried out under the instructions of the two brands of kits. During the execution of each batch of experiments, three negative controls and one weak positive control are required to test. If the test results of negative and positive controls are within the control range, the batch of experiments is valid. Otherwise, it is invalid. Every time laboratory changes reagent batch, 5 samples must be tested with the old lot number reagent. The five samples selected were required to cover the measurement interval, including negative value, critical value, low value, median value, and high value. The document requires that at least 4 of these samples with a measurement bias of less than ±7.5% and the negative and critical value samples must meet expectations. The Ag-RDT assay was performed by the individuals themselves with sample collection strictly following the manufacturer's instructions. After the measurement, the measurement results are uploaded to the system.

### Ethics

The dataset was completely anonymous and did not contain any identifiable personal health information. The study was approved by the Ethics Committee of the First Hospital of Jilin University (No. K2022028).

## Results

### Summary of the study population

A total of 279 individuals met the positive criteria for the initial Ag-RDT screening. After confirmation of positivity by RT-qPCR, 236 screened individuals were included. Ultimately, samples were diagnosed as positive by both Ag-RDT and RT-qPCR, meaning that Ag-RDT positive but RT-qPCR negative was excluded from the study. The age of participants ranged from 1 to 95 years old, with a median age of 48 (IQR, 34–64) years. Of the 236 patients, 109 (46.19%) males with median age of 47 (IQR, 29–59) years and 127 (53.81%) females with median age of 48 (IQR, 32–64) years, were determined to be positive result according to the RT-qPCR.

### Relation to Ct and comparison of kits

**Figure 1:**
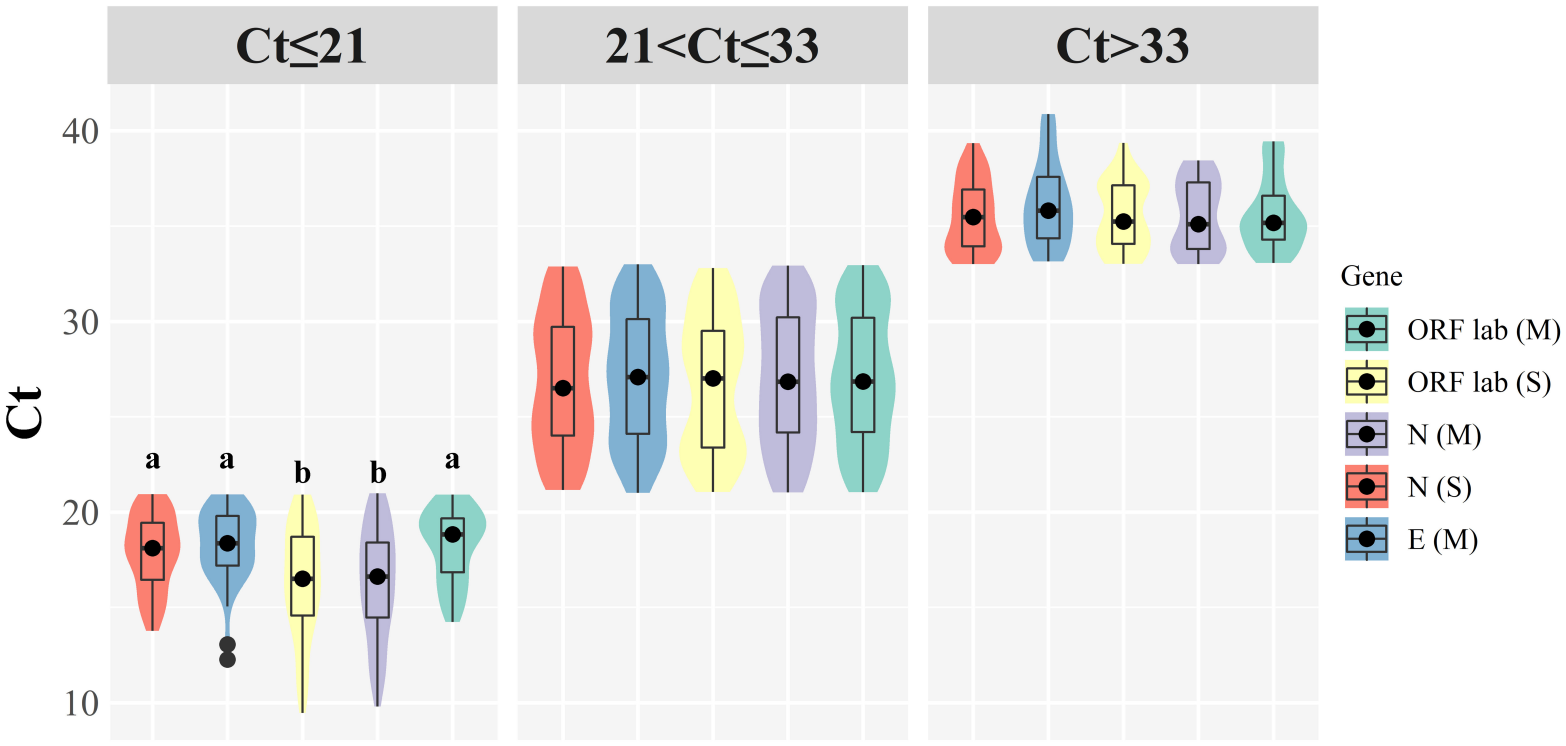
Grouped violin plots for different target genes. (M): Maccura kit, (S): Sansure kit, black dot: median, solid black line: boxplot, groups with significant differences are marked with different letters.

The median of Ct was 25.89 (min Ct 13.77, max Ct 39.35) for the ORF1ab gene, 24.10 (min Ct 9.46, max Ct 39.36) for the N gene, and 26.60 (min Ct 14.23, max Ct 39.45) for the E gene when the Maccura kit used for determination. As for testing with the Sansure kit, the median of Ct was 27.40 (min Ct 12.26, max Ct 40.88) for the ORF1ab gene, 25.20 (min Ct 9.8, max Ct 38.45) for the N gene. When Ct ≤ 21, significant differences were shown among target genes (*P* < 0.05, Table 1). Regardless of the kit type or Ct group, the Ct of N genes demonstrated a lower level than other target genes. In terms of gender and onset time, there were no significant statistical differences in all groups. Grouped violin plots for different target genes are shown in Figure 1.

**Table 1:** Data characteristics of different Ct groups.

**Group**	**Gene**	**Ct**	**Age**
Ct ≤ 21*	ORF lab (M)	18.11 (16.44–19.44)	52 (33–66)
	ORF lab (S)	18.36 (17.19–19.80)	52 (41–66)
	N (M)	16.50 (14.55–18.72)	53 (36–66)
	N(S)	16.62 (14.47–18.40)	52 (33–66)
	E (M)	18.83 (16.84–19.67)	52 (34–65)
21 < Ct ≤ 33	ORF lab (M)	26.50 (24.01–29.71)	47 (27–60)
	ORF lab (S)	27.09 (24.10–30.12)	47 (30–60)
	N (M)	27.02 (23.36–29.59)	44 (25–59)
	N (S)	26.84 (24.15–30.23)	45 (28–59)
	E (M)	26.86 (24.20–30.20)	48 (26–61)
Ct > 33	ORF lab (M)	35.48 (33.90–37.07)	38 (27–51)
	ORF lab (S)	35.82 (34.37–37.59)	37 (22–59)
	N (M)	35.24 (34.07–37.14)	44 (36–59)
	N (S)	35.10 (33.80–37.30)	43 (32–59)
	E (M)	35.18 (34.29–36.59)	43 (30–58)

^*^P < 0.05 (Kruskal-Wallis test), M, Maccura kit; S, Sansure kit.

### Relation to symptoms

**Figure 2:**
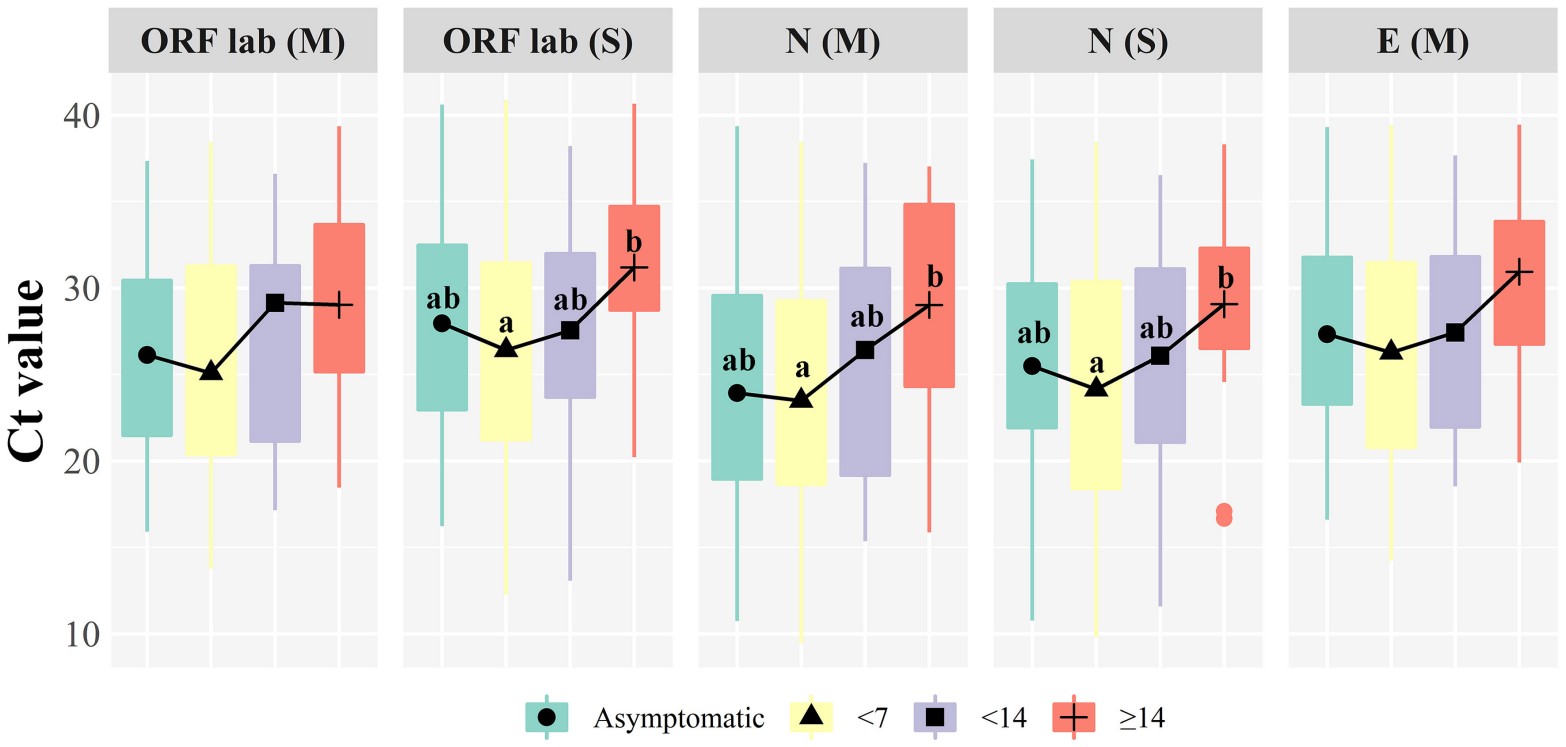
Boxplot of target genes comparison at different onset time. (M): Maccura kit, (S): Sansure kit, groups with significant differences are marked with different letters.

Thirty-five patients were asymptomatic infections, meaning that the RT-qPCR was positive before symptoms appeared. There were 13 males (37.14%) and 22 females (62.86%) in this group of patients, with a median age of 47 years (IQR: 31–59 years). Within 7 days of onset, there were 160 patients with a median age of 48 years (IQR: 31–63 years). At 7–14 days of onset, there were 19 patients with a median age of 50 years (IQR: 37–62 years). Twenty-two patients had an onset for more than 14 days with a median age of 38 years (IQR: 20–58 years). Significant differences were found between the Ct of different target genes in COVID-19 patients with an onset time of 0–7 days (*P* < 0.05, Table 2). Figure 2 illustrates that the N gene presents a lower value among all target genes, and the ORF1ab using the Sansure kit presents a higher measurement value. It was found that the ORF1ab gene determined by Sansure kit and the N gene determined by the Sansure and Maccura kit showed significant differences in different onset times (*P* < 0.05, Table 2) when comparing the onset time of different target genes. Generally, all target genes showed low measured values within 0–7 days, and then the measured Ct steadily increased with time.

**Table 2:** Characteristics of Ct in different onset time periods.

**Gene**	**Asymptomatic** **(*n* = 35, 14.83%)**	**0–7 days** **(*n* = 160, 67.80%)**	**7–14 days** **(*n* = 19, 8.05%)**	**>14 days** **(*n* = 22, 9.32%)**	***P***
ORF lab (M)	26.12 (21.48–30.44)	25.08 (20.37–31.27)	29.14 (21.16–31.28)	29.03 (25.17–33.66)	0.214
ORF lab (S)	27.95 (22.97–32.47)	26.41 (21.19–31.45)	27.55 (23.69–31.98)	31.19 (28.73–34.72)	0.026
N (M)	23.92 (18.73–29.77)	23.48 (18.64–29.26)	26.41 (19.18–31.13)	29.02 (24.32–34.83)	0.049
N (S)	25.49 (21.93–30.23)	24.14 (18.40–30.41)	26.08 (21.08–31.10)	29.07 (26.51–32.36)	0.020
E (M)	27.33 (23.28–31.82)	26.27 (20.74–31.50)	27.43 (21.97–31.80)	30.94 (26.76–33.84)	0.097
*P*	0.207	0.002	0.764	0.765	

M, Maccura kit; S, Sansure kit.

### Methodological comparison

**Figure 3:**
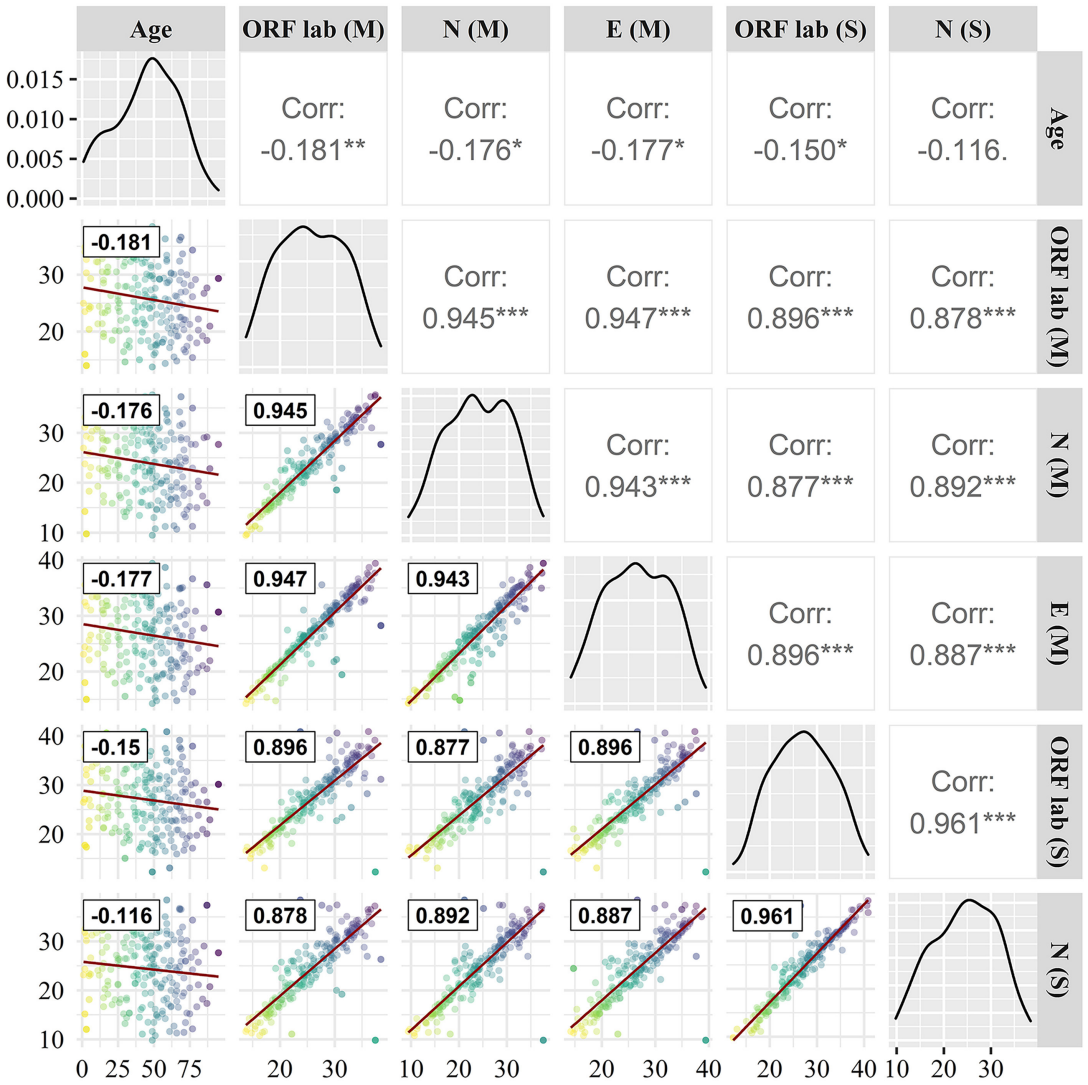
Correlation comparison of target genes. (M): Maccura kit, (S): Sansure kit, red straight line: linear fit curve, black box line: Correlation coefficient. * *P* < 0.05, ** *P* < 0.01, *** *P* < 0.001.

As can be seen from Figure 3, the determination of the same gene by different manufacturers shows strong consistency, the correlation between Maccura and Sansure kit is 0.896 for the ORF1ab (*P* < 0.05), and 0.892 (*P* < 0.05) for the N gene. Strong correlations were displayed among the Ct of the ORF1ab gene, N gene, and E gene, and the correlation coefficients were all >0.870. Moreover, influencing factors do not include age for the determination of the SARS-CoV-2 gene and the correlation between Ct detection and age for different genes was < 0.200 (maximum detection value was 0.181).

## Discussion

Ct in RT-qPCR refers to the moment when amplification occurs and is deemed as a surrogate marker for viral load. This value is inversely proportional to the viral RNA copy number, and a lower Ct means a high accumulation of viral load (10). While RT-qPCR is regarded as the reference method for detecting SARS-CoV-2, SARS-CoV-2 RNA detected by this technique does not indicate the presence or shedding of live replication-competent virus, and whether the person was contagious at the time of the test (11). Studies have shown that the culture-positive rate of samples with Ct between 13 and 17 is 100%. With a Ct of 33, the culture-positive rates drop to 12%, and there is no virus growth with a Ct of ≥34, confirming that patients with these values do not excrete infectious viral particles (12). A study of hospitalized patients with COVID-19 reported that the median time to shed infectious virus was 8 days after symptom onset and the chance of continued shedding of the infectious virus after 15 days was < 5% (13). Of course, it would be unwise to compare Ct from different studies individually, and the correlation between viral load and the risk of transmission from positive cases remains unclear. Nevertheless, it is certain that there is a positive correlation between high viral load and high infectivity (14). In the present study, N genes with low viral load levels exhibited lower Ct, implying that N genes are more sensitive to the discrimination of viral load. The situation may be due to the highly conserved N gene of SARS-CoV-2 and the high relative abundance of N gene subgenomic mRNA produced during viral replication (15). A study demonstrated that N-gene-based RT-qPCR assays are indeed more sensitive than the open reading frame 1 assays in detecting SARS-CoV-2 in clinical specimens (16). Studies have also revealed that primers targeting N2 or E genes have higher sensitivity (17).

The process of viral load and antigenic response is variable and dynamic throughout infection (18). Viral load has been reported to peak (108.1 copies) within 7 days (4.3 days) of onset and subsequently decline at an estimated rate of 0.17 log10 units per day (19). One study confirmed that viral load in respiratory specimens peaked 4 days after symptom onset, followed by a rapid decline, stopping significantly after day 10 in upper respiratory specimens and 15 days in lower respiratory specimens (20). Several studies showed similar viral load trends to the present study, with a significant increase in viral load within 1 week of onset followed by a gradual decline (21, 22). As reviewed by Cevik et al., SARS-CoV-2 viral load appears to peak during the first week of upper respiratory disease (23). N genes measured by kits from different manufacturers showed lower values at different onset times in the current study. The Ct also varied greatly at different stages of onset time, especially for the N gene on 0–7 days. This may be closely related to the high expression for a set of subgenomic RNAs of N gene (24).

Various target genes were measured by two kinds of test kit. The results revealed a good agreement in Ct for the same gene. Test kits for E, N, and ORF1ab gene assays show high sensitivity in other studies evaluating clinical presentations (25). Of course, the core detection genes recommended by different researches are slightly discordance, and there are also inconsistently opinion on the detection sensitivity of target genes. For example, Ramirez et al. emphasized the role of the E gene for the diagnosis of SARS-CoV-2 (26). Combined diagnostics using multiple viral gene panels are recommended to improve assay performance. A comparative analysis study by Jung et al. showed that the combination of ORF1 ab, N2, N3, and NIID display the most sensitive and reliable detection of target fusions (27).

Certainly, there are some limitations to conducting research. For instance, many factors, such as different laboratory measurement instruments, personnel, sample collection quality, storage and transportation conditions, exist in clinical assays. The above reasons directly make it hard for this study to make a comprehensive comparison with other laboratory data.

The diagnosis of COVID-19 determined by the combined application of Ag-RDT and RT-qPCR can provide a rapid understanding of infection in the region, reducing laboratory stress and delays in diagnosis. The study confirmed the determination performance of the N gene at a low Ct and low onset time (0–7 days), and the gene also has a certain ability to discriminate asymptomatic infection. The target genes showed strong concordance. Taken together, this study elucidates the impact of onset time, target genes, and reagent manufacturers on diagnosis by comparing viral loads, helping to identify and control disease epidemics in a timely and effective manner.

## Data availability statement

The raw data supporting the conclusions of this article will be made available by the authors, without undue reservation.

## Ethics statement

The studies involving human participants were reviewed and approved by the Ethics Committee of the First Hospital of Jilin University. Written informed consent from the participants' legal guardian/next of kin was not required to participate in this study in accordance with the national legislation and the institutional requirements.

## Author contributions

XZ, QZ, and JX contributed to the design of the study. FZ were responsible for data collection and collation. XZ and JX wrote and reviewed the manuscript. All authors agreed to be accountable for the content of the work.

## Funding

This work was supported by the Jilin Science and Technology Development Program (Grant numbers 20190304110YY, 20200404171YY), the First Hospital Translational Funding for Scientific and Technological Achievements (Grant number CGZHYD202012-005).

## Conflict of interest

The authors declare that the research was conducted in the absence of any commercial or financial relationships that could be construed as a potential conflict of interest.

## Publisher's note

All claims expressed in this article are solely those of the authors and do not necessarily represent those of their affiliated organizations, or those of the publisher, the editors and the reviewers. Any product that may be evaluated in this article, or claim that may be made by its manufacturer, is not guaranteed or endorsed by the publisher.
